# Supplementary figures and images for: Epigenome Wide Association and Stochastic Epigenetic Mutation Analysis on Cord Blood of Preterm Birth
Source: Int J Mol Sci. 2020 Jul 17;21(14):5044. doi: 10.3390/ijms21145044 (PMC7403978; doi:10.3390/ijms21145044)

CD4T

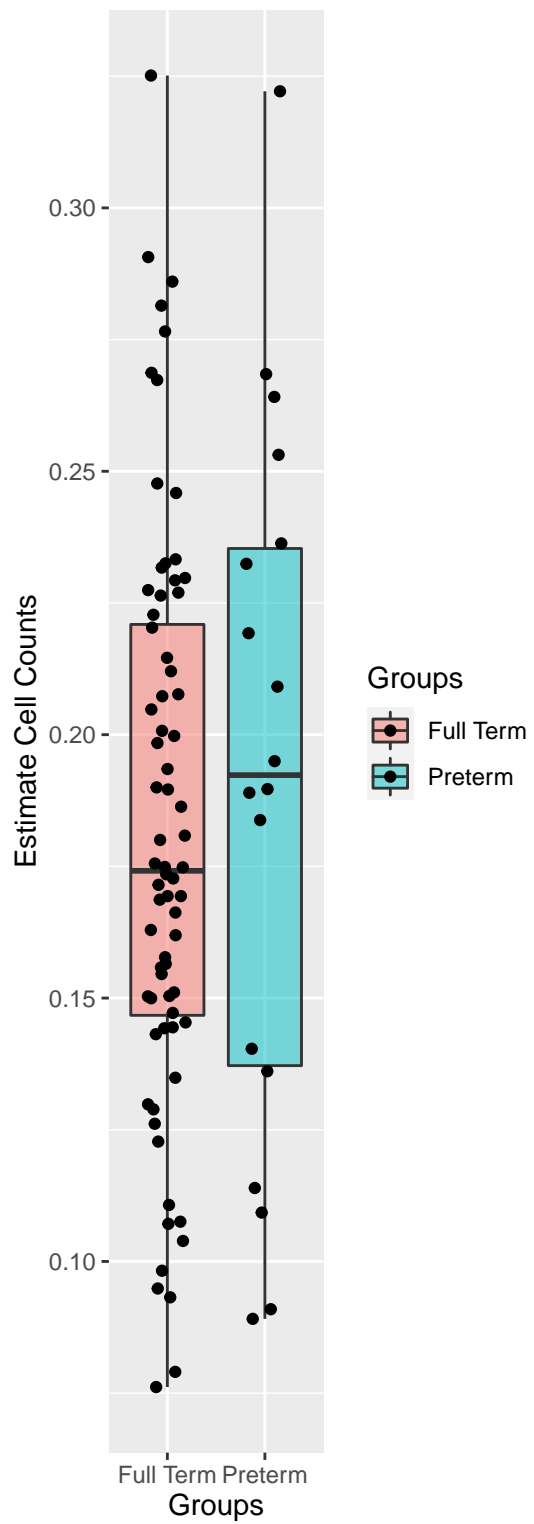

CD8T

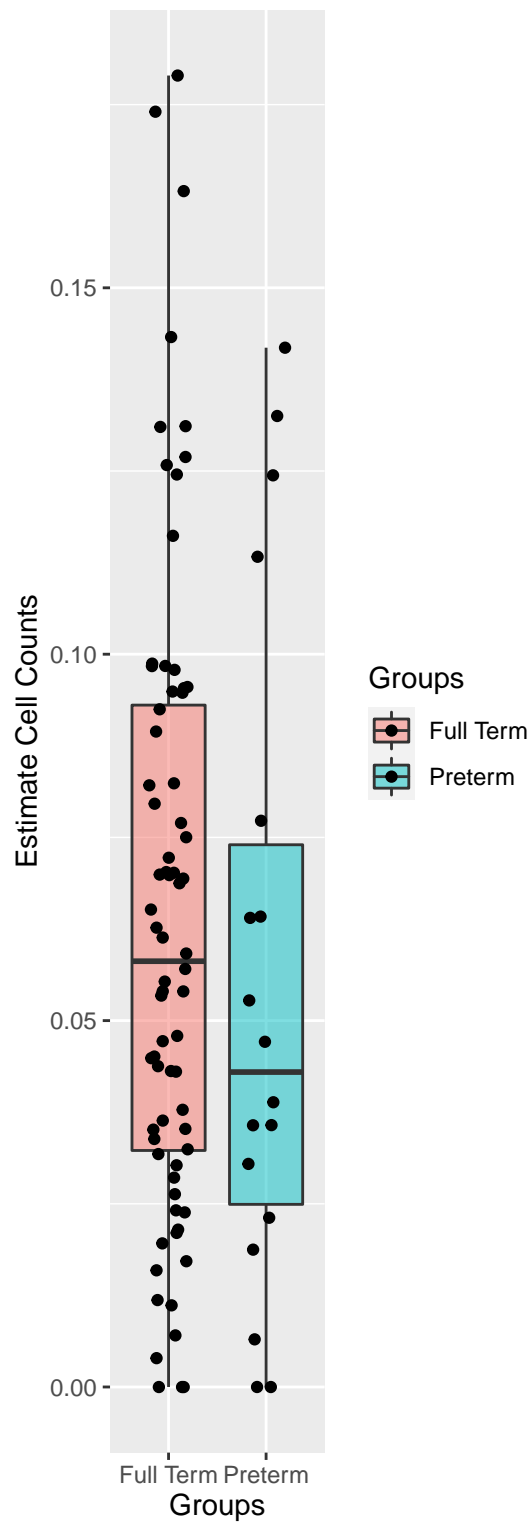

Monocytes

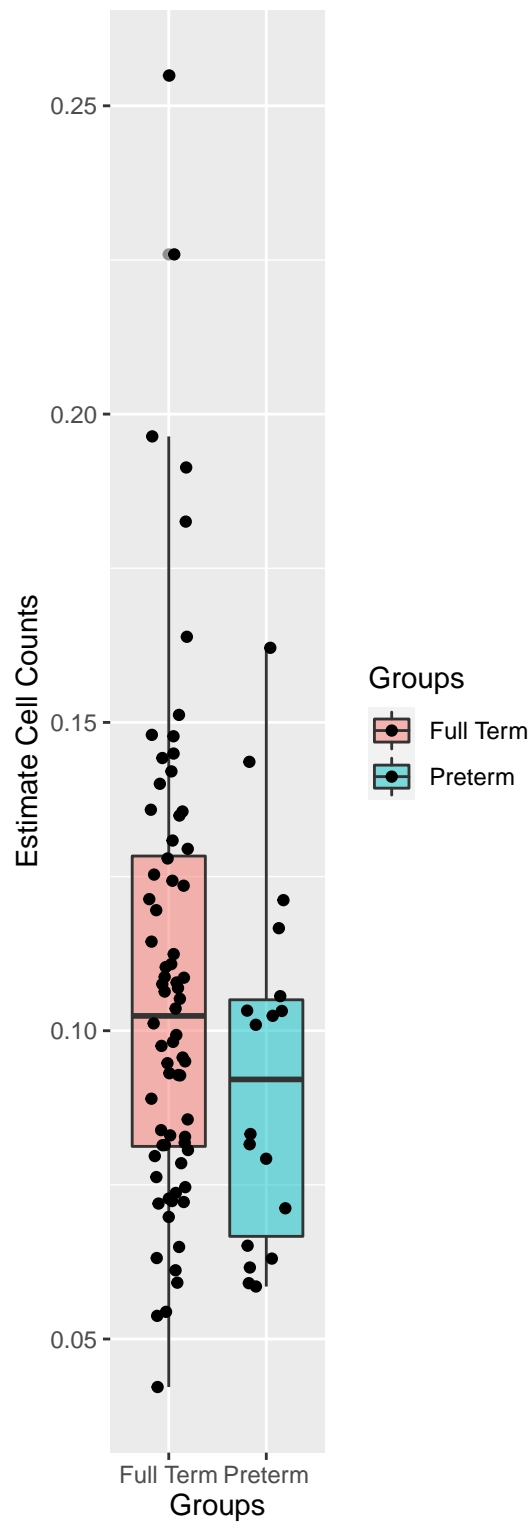

Plasmablasts

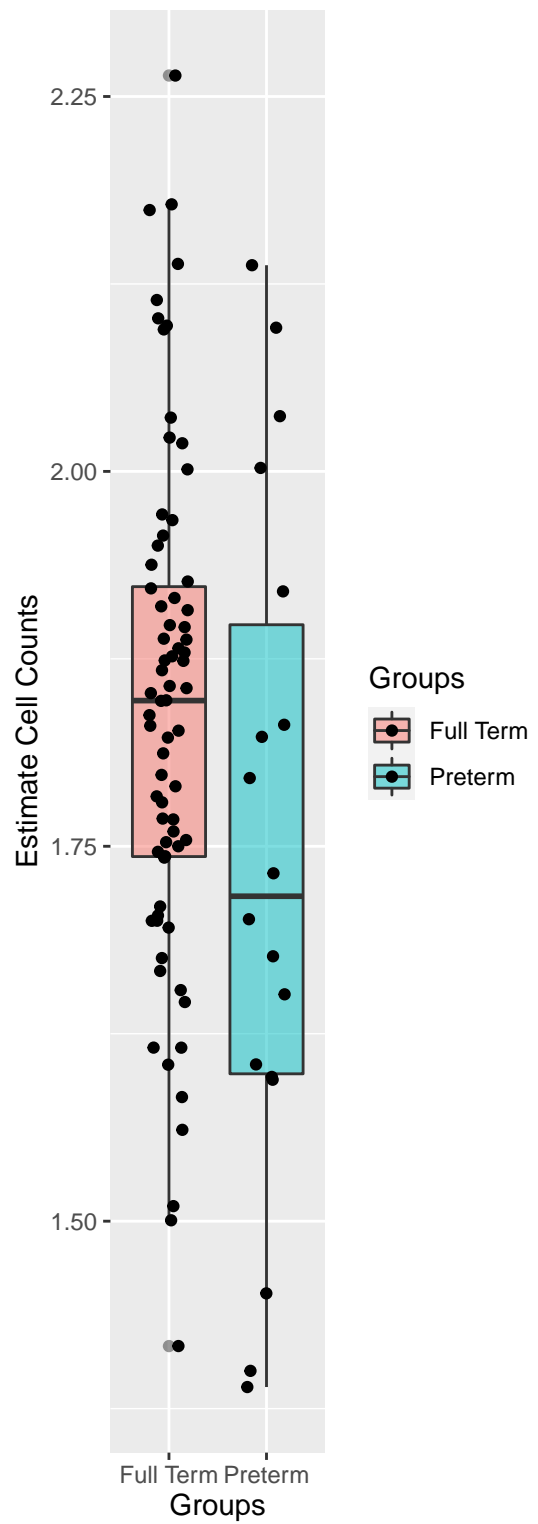

Supplement: Supplementary file 1 [file ijms-21-05044-s001.zip › Supplementary Figure S1.pdf]

REVIGO Gene Ontology treemap

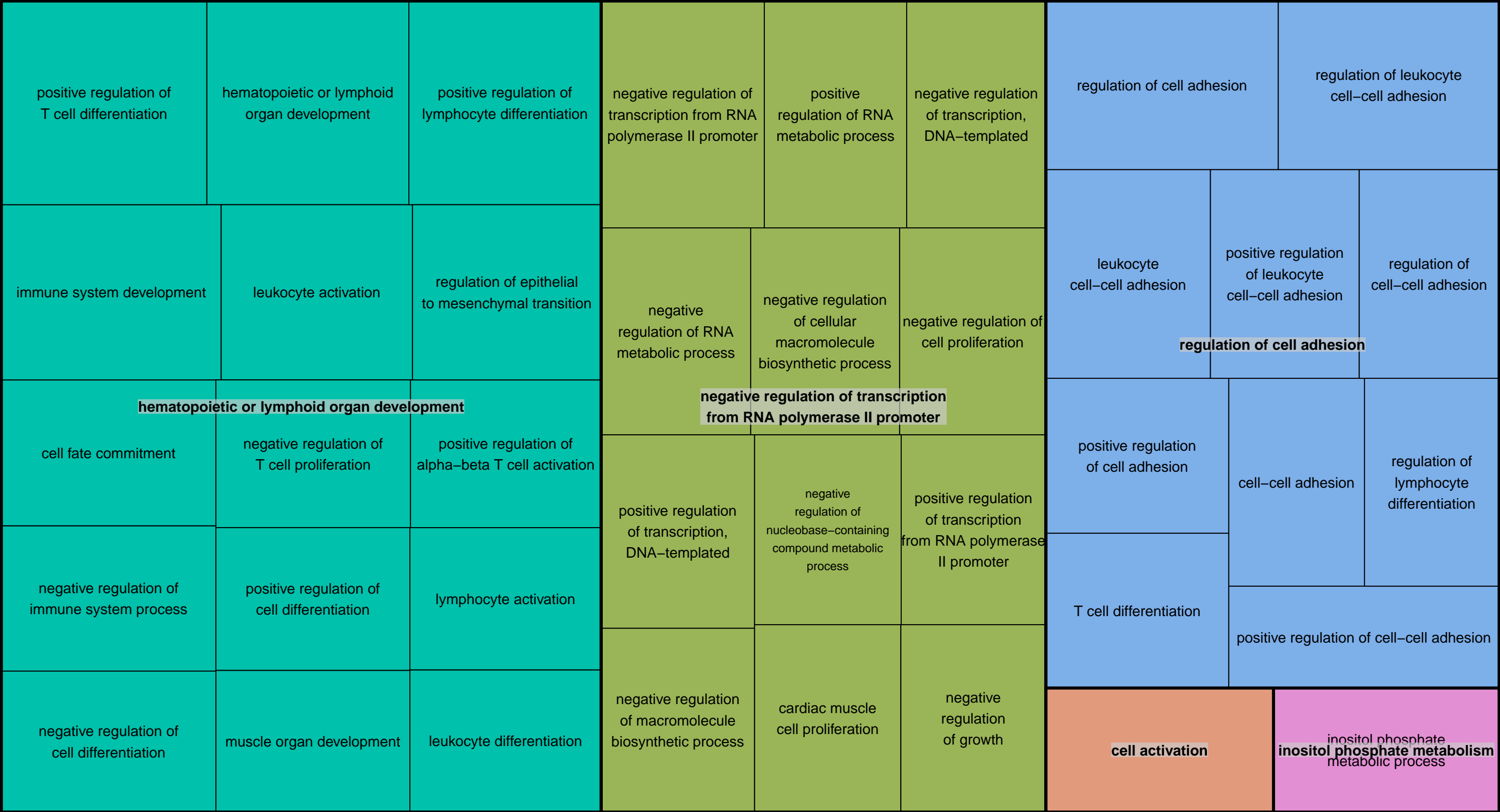

Supplement: Supplementary file 1 [file ijms-21-05044-s001.zip › Supplementary Figure S2.pdf]
